# Supplementary material for: Close spatial arrangement of mutants favors and disfavors fixation
Source: PLoS Comput Biol. 2019 Sep 16;15(9):e1007212. doi: 10.1371/journal.pcbi.1007212 (PMC6746358; doi:10.1371/journal.pcbi.1007212)
Supplement: S1 Appendix — (PDF) [file pcbi.1007212.s004.pdf]

Algorithms for transformation between the state number and the triplet.

Functions *triplet_to_state* and *state_to_triplet* shows the transformations between the triplet and the state.

Algorithm 1 triplet_to_state(n, x, a, b) to map the triplet to the state

Input:
The total individual number N;
The triplet x, a, b;
Output: The state

if $a > b$ **then**
  swap(a, b) // Relation constraint
end if
if $x > n - a - b$ **then**
  $x \leftarrow n - a - b$ // Relation constraint
end if
if $x = 0$ **then**
  return F_{a+b}
end if
if $a = 0$ **then**
  return F_b
end if
$i \leftarrow 0$
for $w \leftarrow 0$ **to** $(a + b)$ **do**
  $i \leftarrow i + \lfloor \frac{w}{2} \rfloor \cdot \lfloor \frac{n-w}{2} \rfloor$
end for
$i \leftarrow i + (a - 1) \cdot \lfloor \frac{n-a-b}{2} \rfloor$
$i \leftarrow i + x - 1$
return S_i

Algorithm 2 state_to_triplet(i) to map the state number to the triplet

Input:
The state number i;
Output: x, a, b

$a \leftarrow 1$
$w \leftarrow 0$
while $i \geq \lfloor \frac{w}{2} \rfloor \cdot \lfloor \frac{n-w}{2} \rfloor$ **do**
  $i \leftarrow i - \lfloor \frac{w}{2} \rfloor \cdot \lfloor \frac{n-w}{2} \rfloor$
  $w \leftarrow w + 1$ // To obtain the number of mutants
end while
while $i \geq \lfloor \frac{n-w}{2} \rfloor$ **do**
  $i \leftarrow i - \lfloor \frac{n-w}{2} \rfloor$
  $a \leftarrow a + 1$
end while
$b \leftarrow w - a$
$x \leftarrow i + 1$
return x, a, b
