# Supplementary material for: Close spatial arrangement of mutants favors and disfavors fixation
Source: PLoS Comput Biol. 2019 Sep 16;15(9):e1007212. doi: 10.1371/journal.pcbi.1007212 (PMC6746358; doi:10.1371/journal.pcbi.1007212)
Supplement: S2 Appendix — (PDF) [file pcbi.1007212.s005.pdf]

The algorithm to obtain the transition matrices.

Algorithm 1 shows how to obtain the transition matrices Q_1 and Q_2. There are at most 8 operations for each state. As there are totally $O(N^2)$ states, the time complexity of the algorithm is $O(N^2)$. And the space complexity of the algorithm are the same as the scale of the matrix Q_1, which is $O(N^4)$. It is noteworthy that Q_1 and Q_2 are sparse matrices when N is great. Therefore, the space complexity is $O(N^2)$ if sparse storing methods are adopted.

Algorithm 1 Calculation of Q_1 and Q_2

Input:
Total individual number N;
Mutant payoff r;
Output: Q_1, Q_2

$Total \leftarrow 0$ // The number of the states
for $w \leftarrow 0$ **to** $N+1$ **do**
    $Total \leftarrow Total + \lfloor \frac{w}{2} \rfloor \cdot \lfloor \frac{n-w}{2} \rfloor$
end for
$Q_1 \leftarrow [0]_{Total \times Total}$
$Q_2 \leftarrow [0]_{Total \times (N+1)}$
for $i \leftarrow 1$ **to** $Total$ **do**
    $prob\_self \leftarrow 1$ // The probability not to transit to another state
    for $j \leftarrow 1$ **to** 11 **do**
        $x, a, b \leftarrow state\_to\_triplet(i)$
        Change x, a, b according to Table 2
        $t \leftarrow triplet\_to\_state(N, x, a, b)$
        Assign $temp\_prob$ according to Table 2
        if $t \in F$ **then**
            $Q_2[S_i, t] \leftarrow Q_2[S_i, t] + temp\_prob$
        else
            $Q_1[S_i, t] \leftarrow Q_1[S_i, t] + temp\_prob$
        end if
        $prob\_self \leftarrow prob\_self - temp\_prob$
    end for
    $Q_1[S_i, S_i] \leftarrow Q_1[S_i, S_i] + prob\_self$
end for
return Q_1, Q_2
