# Supplementary material for: Close spatial arrangement of mutants favors and disfavors fixation
Source: PLoS Comput Biol. 2019 Sep 16;15(9):e1007212. doi: 10.1371/journal.pcbi.1007212 (PMC6746358; doi:10.1371/journal.pcbi.1007212)
Supplement: S3 Appendix — (PDF) [file pcbi.1007212.s006.pdf]

The first-order and second-order derivatives of fixation probability.
In the following, we give the details of calculating the first-order derivatives and the second-order derivatives of the fixation probability Ψ. In general, we have

$$\Psi(r) = [I - Q_1(r)]^{-1} Q_2 \pi(r). \tag{1}$$

Where I is an identity matrix of the same size with Q_1. The derivation of Ψ is

$$\frac{d}{dr}\Psi(r) = \frac{d}{dr}[I - Q_1(r)]^{-1} Q_2 \pi(r) + [I - Q_1(r)]^{-1} Q_2 \frac{d}{dr}\pi(r). \tag{2}$$

Since we have the analytical result for $\pi(r)$, the second part of the right side of equation is acknowledged. The point of the problem comes to $\frac{d}{dr}[I - Q_1(r)]^{-1}$.
The property of the matrix inversion gives rise to

$$[I - Q_1(r)]^{-1}[I - Q_1(r)] = I. \tag{3}$$

The derivations of both sides of the equation is

$$\frac{d}{dr}[I - Q_1(r)]^{-1}[I - Q_1(r)] + [I - Q_1(r)]^{-1}\frac{d}{dr}[I - Q_1(r)] = 0. \tag{4}$$

This is equivalence to

$$\frac{d}{dr}[I - Q_1(r)]^{-1}[I - Q_1(r)] = [I - Q_1(r)]^{-1}\frac{d}{dr}Q_1(r). \tag{5}$$

Multiply $[I - Q_1(r)]^{-1}$ for both sides, we have

$$\frac{d}{dr}[I - Q_1(r)]^{-1} = [I - Q_1(r)]^{-1}\frac{d}{dr}Q_1(r)[I - Q_1(r)]^{-1}. \tag{6}$$

Taking Eq. (6) into Eq. (2) results in

$$\frac{d}{dr}\Psi(r) = [I - Q_1(r)]^{-1}\frac{d}{dr}Q_1(r)[I - Q_1(r)]^{-1} Q_2 \pi(r) + [I - Q_1(r)]^{-1} Q_2 \frac{d}{dr}\pi(r), \tag{7}$$

And the second-order derivative can be obtained similarly.
